# UNDERSTANDING RISK AND PROTECTIVE FACTORS FOR ELDER FAMILY FINANCIAL EXPLOITATION: A PREVENTIVE APPROACH

**DOI:** 10.1093/geroni/igz038.694

**Published:** 2019-11-08

**Authors:** Virginia B Vincenti, Ashton Chapman

**Affiliations:** 1 University of Wyoming, Laramie, Wyoming, United States; 2 Social Grove, Inc., Joplin, Missouri, United States

## Abstract

This paper presents qualitative data from a phenomenological study of 10 men and 38 women (N=48) within families with designated power of attorney (POA) agents. Participants were from 18 states nationwide, aged 20 to 73 (M = 46.6), and had varying educational and income levels. Eighteen individuals alleged that EFFE occurred in their families, 27 did not, and three didn’t know. The study explored EFFE risk and protective factors within Bronfenbrenner’s PPCT model (Tudge, 2018). Data were analyzed using a thematic, inductive approach. Specifically, person characteristics (e.g. perpetrators’ personality and victims’ cognitive functioning), proximal processes (e.g. family patterns of communication and resource sharing), context (e.g. geographic location), and time (e.g. prevailing legal, economic, and cohort factors) were associated with EFFE recognition and intervention. Implications and applications of the PPCT model for helping professionals (e.g., practitioners such as healthcare providers, attorneys, therapists) will be discussed to raise awareness of risk and protective factors within families that may increase or decrease the likelihood that elder financial exploitation by family members will occur. The goal is to improve prevention through helping families address risk factors before older relatives become dependent, encouraging more proactive planning in development of POA and other end-of-life documents, and detect exploitation early.

